# Supplementary material for: ADAM17 Mediates Proteolytic Maturation of Voltage-Gated Calcium Channel Auxiliary α2δ Subunits, and Enables Calcium Current Enhancement
Source: Function (Oxf). 2022 Mar 17;3(3):zqac013. doi: 10.1093/function/zqac013 (PMC9016415; doi:10.1093/function/zqac013)
Supplement: zqac013_Supplemental_Figures [file zqac013_supplemental_figures.pdf]

SUPPLEMENTAL INFORMATION

Identification that ADAM17 mediates proteolytic maturation of $\alpha_2\delta$-1, and enables calcium current enhancement

Ivan Kadurin[1], Shehrazade Dahimene[1], Karen M Page[1], Joseph I. J. Ellaway[1], Kanchan Chaggar[1], Linda Troeberg[2], Hideaki Nagase[3] and Annette C. Dolphin[1]

Supplemental Figures

Figure S1 (relates to Figure 2)

Cleavage of $\alpha_2\delta$-1 is reduced in DRMs from CRISPR *ADAM17*$^{-/-}$ / *ADAM10*$^{-/-}$ compared to CRISPR WT cells

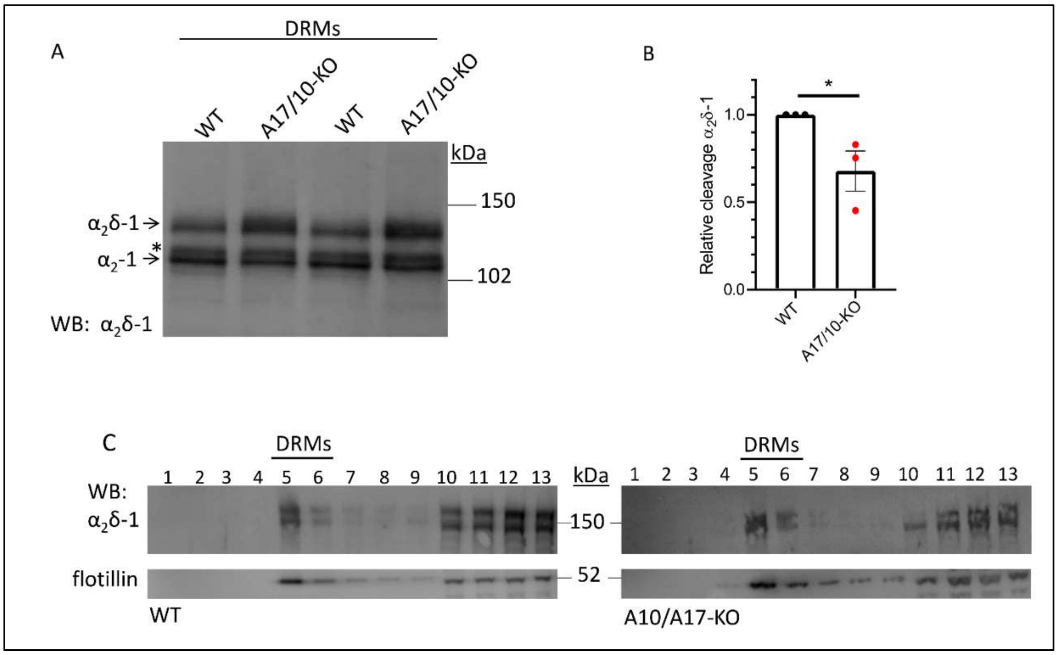

(A) Effect of expression of $\alpha_2\delta$-1 in CRISPR WT (lanes 1 and 3) compared to *ADAM17*$^{-/-}$ / *ADAM10*$^{-/-}$ (A17/10-KO) HEK293 cells (lanes 2 and 4) on cleavage of HA-$\alpha_2\delta$-1 in DRM fraction (α_2-1 immunoblot), de-glycosylated to allow resolution between pro-$\alpha_2\delta$-1 (upper band) and the cleaved form, α_2-1 (lower band). * indicates an intermediate species, which may represent cleavage of $\alpha_2\delta$-1 at an alternative site, or an intermediate product.

(B) Quantification of the effect of expression in *ADAM17*$^{-/-}$ / *ADAM10*$^{-/-}$ cells on relative cleavage of $\alpha_2\delta$-1 in DRMs (normalized to that under control conditions). The data are mean ± SEM and individual data from 3 separate experiments. Statistical difference determined using Student's t

test; * $P = 0.049$. In this study there was ~70% absolute cleavage of $\alpha_2\delta$-1 in DRMs from CRISPR WT cells and ~45% cleavage in *ADAM17*$^{-/-}$ / *ADAM10*$^{-/-}$ cells.

(C) Sucrose gradient profiles (lanes 1-13) showing $\alpha_2\delta$-1 distribution (upper panels) from CRISPR WT (left) compared to *ADAM17*$^{-/-}$ / *ADAM10*$^{-/-}$ HEK293 cells (right). Peak DRM fractions (5 and 6) shown by bar, and by presence of flotillin (lower panels).

Figure S2 (relates to Figure 5)

Most $\alpha_2\delta$-1 in WCL is Endo-H-sensitive in both CRISPR WT and *ADAM17*$^{-/-}$ cells

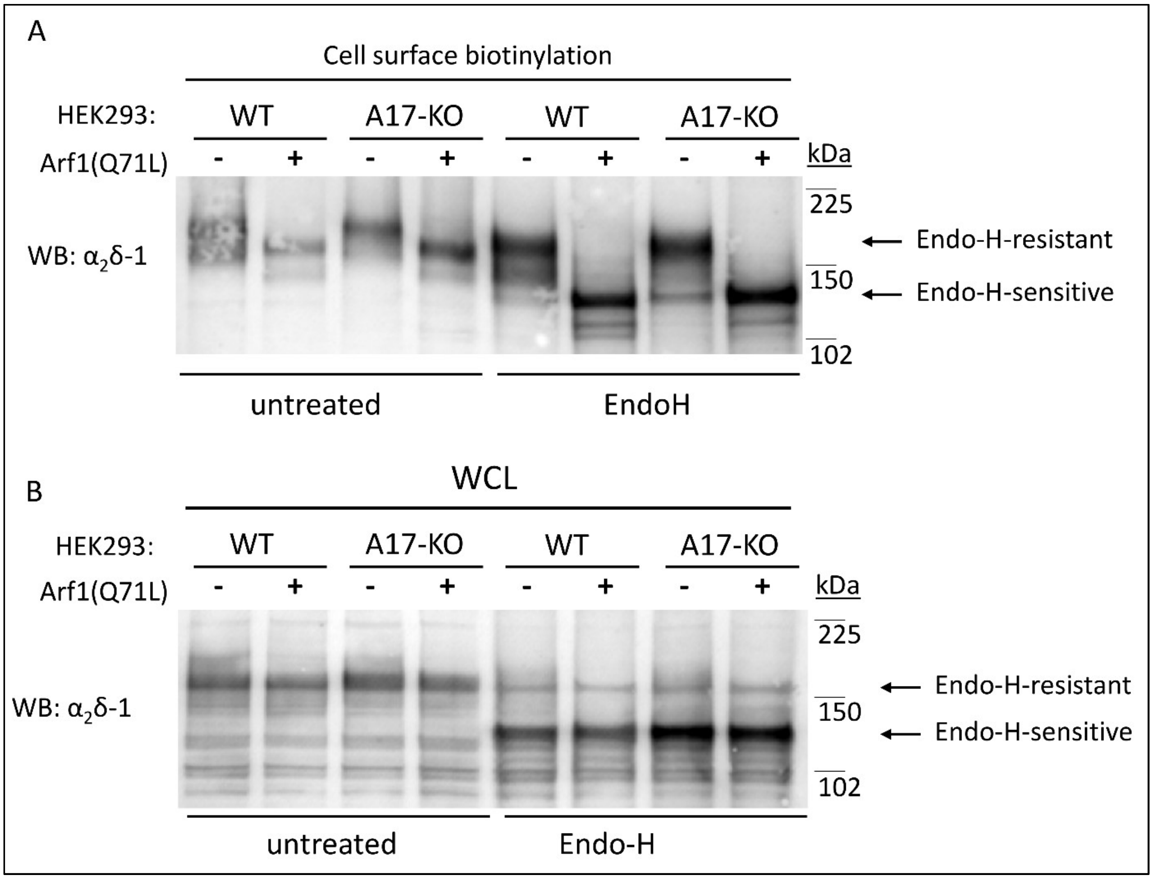

(A, B) Effect of expression of $\alpha_2\delta$-1 in CRISPR WT (lanes 1, 2, 5, 6) and *ADAM17*$^{-/-}$ (A17-KO) cells (lanes 3, 4, 7, 8) in the absence (lanes 1, 3, 5, 7) and presence (lanes 2, 4, 6, 8) of the ER-to-Golgi blocker, Arf1(Q71L), in cell surface biotinylated (A) and WCL (B) samples either left untreated (lanes 1-4) or treated with Endo-H (lanes 5-8) for comparison. The sizes of the Endo-H-resistant bands and Endo-H-sensitive bands are indicated with arrows. Most $\alpha_2\delta$-1 in the WCL is Endo-H-sensitive (lower band), suggesting that it is in the ER and has not yet progressed through the Golgi complex.

Figure S3 (relates to Figure 5)

Gradient centrifugation of $\alpha_2\delta$-3 stable cell line shows proteolytic cleavage appears associated with the Golgi fraction

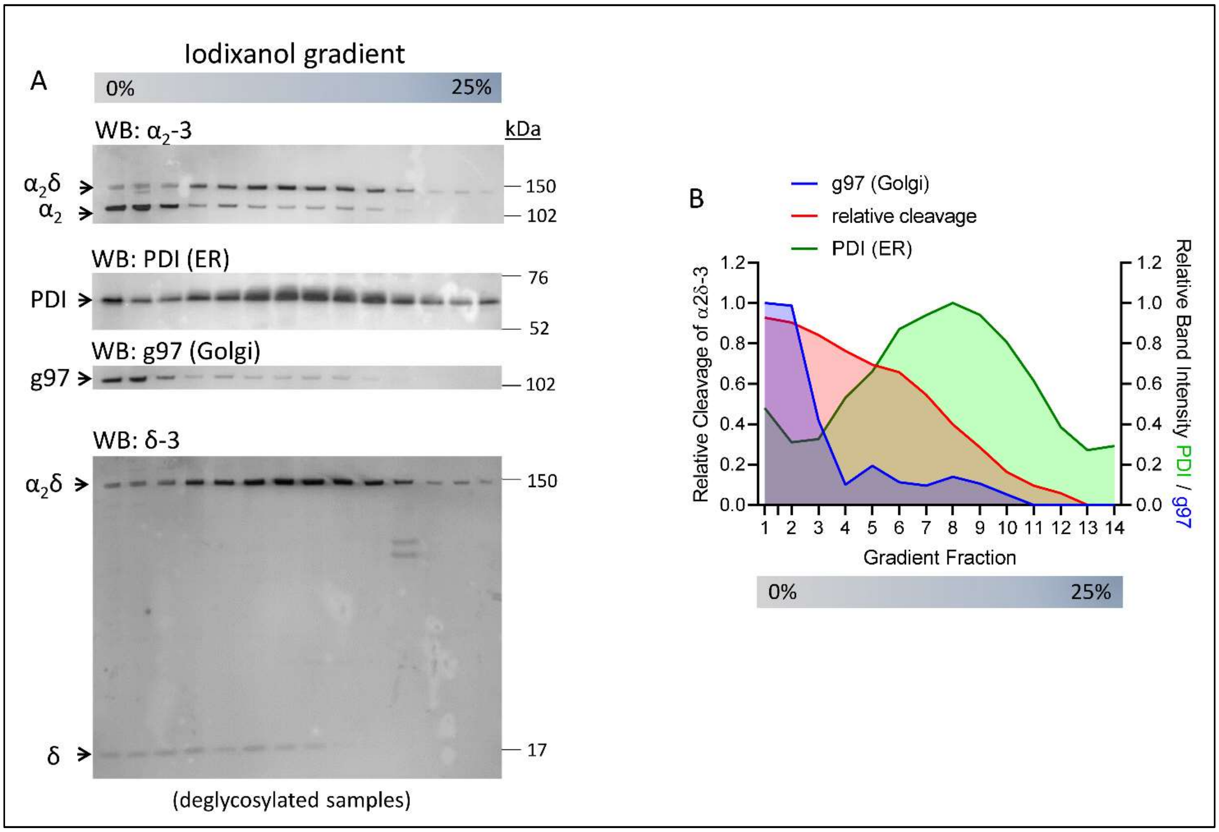

(A) Distribution of $\alpha_2\delta$-3 (α_2-3 Ab, top panel, showing uncleaved $\alpha_2\delta$-3 and cleaved α_2-3, as indicated) following iodixanol gradient centrifugation of SH-SY5Y cells, compared to the ER marker Protein disulfide isomerase (PDI, second panel) and the Golgi marker Golgin 97 (g97, third panel) and δ-3 (bottom panel, showing uncleaved $\alpha_2\delta$-3 and cleaved δ-3, as indicated).

(B) Plot of relative cleavage of $\alpha_2\delta$-3 (red) compared to distribution of g97 (purple) and PDI (green) along iodixanol gradient.
